# Association of Pulse Pressure Index With Mortality in Patients With Hypertension: Results From NHANES 1999–2018

**DOI:** 10.1111/jch.70004

**Published:** 2025-01-29

**Authors:** Hongjin Jin, Shusheng Fang, Shuo An, Yanchun Ding

**Affiliations:** ^1^ Department of Cardiology The Second Hospital of Dalian Medical University Dalian China; ^2^ Department of Critical Care Medicine The Second Hospital of Dalian Medical University Dalian China

**Keywords:** pulse pressure index, pulse pressure, hypertension, all‐cause mortality, cardiovascular mortality

## Abstract

Vascular compliance is an important predictor of cardiovascular disease and mortality. Pulse pressure index (PPI) is a reliable indicator for evaluating vascular compliance. However, the association between PPI, all‐cause mortality (ACM), and cardiovascular mortality (CVM) in patients with hypertension is still unclear. In this study, we aimed to investigate the association of PPI with ACM and CVM in patients with hypertension. Kaplan–Meier survival curves, Cox proportional hazards regression models, restricted cubic splines, and subgroup and interaction analyses were used to investigate the association of PPI with ACM and CVM. U‐shaped associations were observed between PPI and both ACM and CVM, and the inflection points for ACM and CVM were at PPI values of 0.327 and 0.363, respectively. Time‐dependent receiver operating characteristic curves indicated that PPI showed good predictive value for both ACM and CVM occurrence at 1, 3, 5, and 10 years, and its predictive value was higher than PP for ACM and CVM at 5 and 10 years. These results showed that PPI can be used to identify patients with hypertension who are at a high risk of mortality and can guide more aggressive anti‐hypertensive treatment strategies. Moreover, these findings demonstrate that PPI is a superior vascular compliance indicator than PP.

## 1 Introduction

Hypertension is a major global public health challenge and the primary contributor to the worldwide burden of disease and mortality [1]. As of 2019, approximately 1.278 billion individuals aged 30–79 years had hypertension, twice the number from 1990 [2]. Hypertension is the most common preventable risk factor for cardiovascular disease [3], stroke [4], and chronic kidney disease [5]. It leads to varying degrees of target organ damage (TOD) in the heart, brain, and kidneys, increasing the risk of cardiovascular mortality (CVM) and all‐cause mortality (ACM) [6]. Monitoring blood pressure is crucial for delaying TOD and reducing hypertension‐related mortality [7]. Thus, an easily measurable and validated prognostic parameter is imperative.

Pulse pressure (PP) is the difference between systolic blood pressure (SBP) and diastolic blood pressure (DBP), serving as a reliable indicator of arterial vascular compliance [8, 9]. The predictive value of PP in cardiovascular outcomes is widely recognized. The Framingham Heart Study showed that with every 10 mm Hg increase in PP, the risk of coronary artery disease (CAD) increased by 23% [10]. Furthermore, a meta‐analysis indicated that higher PP correlated with an increased risk of ACM and CVM [11]. High PP is an independent risk factor for CVM, even in individuals with normal blood pressure [12]. However, considering the alterable and “floating” characteristics of PP, Yang et al. [13] proposed the “pulse pressure index” (PPI) based on the elastic chamber theory. PPI, which is the ratio of PP to SBP, is negatively correlated with vascular compliance and may be superior to PP in evaluating cardiovascular outcomes [13]. PPI is associated with CAD [14], stroke [15], and other adverse outcomes caused by hypertension.

However, no previous studies have examined the correlation between PPI and mortality in patients with hypertension. Therefore, this study aims to investigate the association of PPI with ACM and CVM in patients with hypertension using data from the National Health and Nutrition Examination Survey (NHANES).

## 2 Methods

### 2.1 Study Population

This study utilized data from the NHANES, an ongoing, independent, nationally representative cross‐sectional survey of the non‐institutionalized civilian population in the United States [16]. The NHANES was approved by the National Center for Health Statistics Research Ethics Review Board, and all participants provided written informed consent.

**FIGURE 1:**
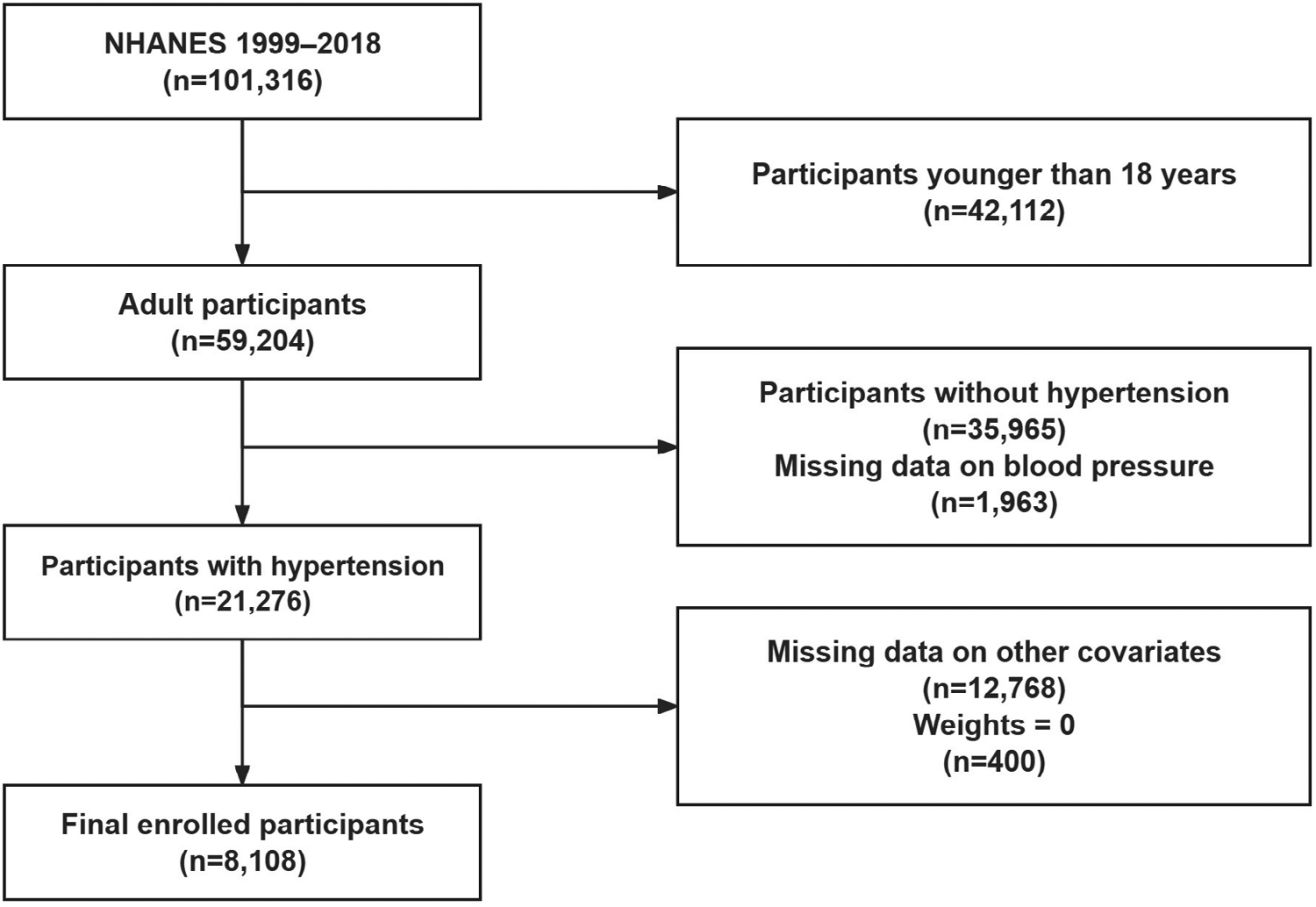
Flowchart of participant selection from NHANES 1999–2018.

We used data from 10 survey cycles of the NHANES (1999–2018), which included 101 316 participants. After excluding participants younger than 18 years old (*n* = 42 112), those without hypertension (*n* = 35 965), those with missing data on blood pressure (*n* = 1 963), those with missing data on other covariates (*n* = 12 768), and those with weights equal to 0 (*n* = 400), a final cohort of 8108 participants with hypertension was included in this study (Figure 1).

### 2.2 Blood Pressure Measurement

Blood pressure was measured by a certified medical professional using a mercury sphygmomanometer at a mobile examination center. Measurements were taken with participants seated, primarily from the right arm, unless special circumstances required otherwise. Before this measurement, upper arm circumference was measured to guide cuff size selection. Following 5 min of rest in a sitting position and determination of the maximum inflation level, blood pressure was measured three consecutive times, with SBP and DBP readings recorded [17]. PP was defined as the difference between SBP and DBP, and PPI was defined as the ratio of PP to SBP. Hypertension was defined by any of the following criteria: (1) SBP ≥ 140 mm Hg, (2) DBP ≥ 90 mm Hg, (3) a self‐reported history of hypertension, or (4) the use of antihypertensive medications [18].

### 2.3 Ascertainment of Mortality

We matched the NHANES data with the linked mortality files provided by the National Center for Health Statistics. The mortality of participants was tracked up to December 31, 2019. ACM was defined as death from any cause. Specific causes of death were determined using the International Classification of Diseases (ICD), 10th Revision. CVM was defined using ICD codes I00–I09, I11, I13, and I20–I51 [19].

### 2.4 Covariates

We screened for covariates based on traditional risk factors for hypertension. Participant characteristics included age, sex, race, education level, body mass index (BMI), smoking history, drinking history, SBP, DBP, PP, PPI, fasting plasma glucose (FPG), hemoglobin type A1C (HbA1c), total cholesterol (TC), total triglycerides (TG), low‐density lipoprotein cholesterol (LDL‐C), and high‐density lipoprotein cholesterol (HDL‐C). Race was categorized as “Mexican American,” “Non‐Hispanic Black,” “Non‐Hispanic White,” “Other Hispanic,” or “Other Race” [20]. Education level was categorized as “Less than high school,” “High school or equivalent,” or “More than high school” [21]. BMI was calculated by dividing weight (kg) by height (m) squared and categorized as normal weight (<25 kg/m^2^), overweight (25 ≤ BMI < 30 kg/m^2^), or obesity (≥30 kg/m^2^) [22]. Smoking history was categorized as “no” (<100 cigarettes in a lifetime) or “yes” (≥100 cigarettes in a lifetime) [23]. Drinking history was categorized as “no” (<12 alcoholic drinks in a lifetime) or “yes” (≥12 alcoholic drinks in a lifetime). Participants were classified as having diabetes if they met any of the following criteria: (1) FPG ≥ 7.0 mmol/L, (2) HbA1c ≥ 6.5%, (3) self‐reported history of diabetes, or (4) current use of hypoglycemic agent or insulin [24].

### 2.5 Statistical Analysis

Given that NHANES is a multistage, stratified, probability‐based survey, all analyses were weighted using the fasting subsample weights (WTSAF4YR and WTSAF2YR) to ensure national representativeness [25]. Participants were categorized into four groups based on their PPI quartiles. Continuous variables were expressed as mean and standard deviation, while categorical variables were presented as the number of cases and the percentage of each category. One‐way analysis of variance (for continuous variables) and Chi‐square tests (for categorical variables) were used to compare the differences in baseline characteristics among the four groups. Kaplan–Meier survival curves were plotted, and the log‐rank test was used to compare the differences in survival probabilities among the four groups. We used Cox proportional hazards regression models to assess the independent association of PPI with ACM and CVM and to calculate hazard ratios (HR) and 95% confidence intervals (CI). Three Cox proportional hazards regression models were used to adjust for the covariates: Model I was unadjusted; Model II adjusted for age, sex, race, education level, BMI category, smoking history, and drinking history; and Model III additionally adjusted for FPG, HbA1c, TC, TG, HDL‐C, and LDL‐C. Restricted cubic spline (RCS) analysis was used to explore the potential non‐linear relationships between PPI and both ACM and CVM. If a non‐linear relationship was observed, threshold effects analysis was conducted to estimate the inflection point. Subsequently, we constructed a two‐piecewise Cox proportional hazards regression model on either side of the inflection point and compared it to the standard Cox proportional hazards regression model using the log‐likelihood ratio test [26]. Subgroup and interaction analyses were conducted according to age (< = 60 and >60 years), sex (Female/Male), smoking history (yes/no), drinking history (yes/no), BMI category (Normal weight, Overweight, and Obesity), and diabetes (yes/no) to assess the effect of PPI on ACM and CVM across different subgroups. Time‐dependent receiver operating characteristic (ROC) curves were used to evaluate the predictive value of PPI for mortality at different time points and to verify if this predictive value was higher than that of PP [27]. Statistical analysis was conducted using R (version 4.3.3) software, with statistical significance set at *p* < 0.05.

## 3 Results

### 3.1 Baseline Characteristics

Our study included 8108 participants with hypertension, exhibiting a mean age of 59.88 years and a male representation of 48.9%. These participants were categorized into four groups based on their PPI quartiles. Compared to those in the first quartile, participants in the fourth quartile exhibited several distinguishing characteristics: they were older, more likely to be female, more likely to be Non‐Hispanic White, less educated, had a lower BMI, less likely to have a history of drinking, and more likely to have a history of diabetes. Additionally, their blood pressure profile was characterized by higher SBP, lower DBP, and higher PP. Moreover, participants in this quartile were at a higher risk of ACM and CVM (*p* < 0.05). Further baseline characteristics of the participants are shown in Table 1.

**TABLE 1:** Baseline characteristics of participants with hypertension based on PPI quartiles.

Characteristic	Total (*n* = 8108)	Q1 (<0.39) (*n* = 2027)	Q2 (0.39–0.45) (*n* = 2027)	Q3 (0.45–0.53) (*n* = 2027)	Q4 (>0.53) (*n* = 2027)	*p*
**Age, years**	59.88(15.30)	49.44(12.77)	56.26(13.93)	63.34(13.75)	70.47(12.00)	<0.0001
**Sex, *n* (%)**						<0.0001
Female	4143(51.1)	890(43.9)	991(48.9)	1054(52.0)	1208(59.6)	
Male	3965(48.9)	1137(56.1)	1036(51.1)	973(48.0)	819(40.4)	
**Race, *n* (%)**						<0.0001
Mexican American	1143(14.1)	249(12.3)	290(14.3)	312(15.4)	292(14.4)	
Non‐Hispanic Black	1917(23.6)	496(24.5)	542(26.7)	495(24.4)	384(18.9)	
Non‐Hispanic White	3849(47.5)	949(46.8)	883(43.6)	930(45.9)	1087(53.6)	
Other Hispanic	617(7.6)	149(7.4)	153(7.5)	150(7.4)	165(8.1)	
Other Race	582(7.2)	184(9.1)	159(7.8)	140(6.9)	99(4.9)	
**Education level, *n* (%)**						<0.0001
Less than high school	2347(28.9)	433(21.4)	552(27.2)	630(31.1)	732(36.1)	
High school or equivalent	2023(25.0)	492(24.3)	463(22.8)	564(27.8)	504(24.9)	
More than high school	3738(46.1)	1102(54.4)	1012(49.9)	833(41.1)	791(39.0)	
**BMI category, *n* (%)**						<0.0001
Normal weight	1659(20.5)	321(15.8)	356(17.6)	444(21.9)	538(26.5)	
Overweight	2692(33.2)	625(30.8)	653(32.2)	694(34.2)	720(35.5)	
Obesity	3757(46.3)	1081(53.3)	1018(50.2)	889(43.9)	769(37.9)	
**Smoking history, *n* (%)**						0.1594
NO	4067(50.2)	1042(51.4)	1027(50.7)	974(48.1)	1024(50.5)	
YES	4041(49.8)	985(48.6)	1000(49.3)	1053(51.9)	1003(49.5)	
**Drinking history, *n* (%)**						<0.0001
NO	1226(15.1)	237(11.7)	280(13.8)	303(14.9)	406(20.0)	
YES	6882(84.9)	1790(88.3)	1747(86.2)	1724(85.1)	1621(80.0)	
**Diabetes, *n* (%)**						<0.0001
NO	5867(72.4)	1601(79.0)	1522(75.1)	1426(70.4)	1318(65.0)	
YES	2241(27.6)	426(21.0)	505(24.9)	601(29.6)	709(35.0)	
**SBP (mm Hg)**	136.68(20.67)	124.85(15.82)	132.28(17.28)	140.30(18.91)	149.29(21.74)	<0.0001
**DBP (mm Hg)**	72.68(13.69)	83.32(10.65)	76.49(10.04)	71.46(9.77)	59.43(11.63)	<0.0001
**PP (mm Hg)**	64.01(21.28)	41.53(8.14)	55.80(8.04)	68.84(10.09)	89.86(17.75)	<0.0001
**PPI**	0.46(0.11)	0.33(0.04)	0.42(0.02)	0.49(0.02)	0.60(0.06)	<0.0001
**FPG (mmol/L)**	6.36(2.10)	6.16(1.97)	6.29(2.15)	6.48(2.13)	6.51(2.12)	<0.0001
**HbA1c, %**	5.96(1.15)	5.80(1.14)	5.92(1.15)	6.01(1.15)	6.09(1.16)	<0.0001
**TC (mmol/L)**	5.05(1.09)	5.11(1.06)	5.09(1.07)	5.01(1.08)	4.99(1.14)	0.0005
**TG (mmol/L)**	1.50(0.79)	1.54(0.82)	1.50(0.79)	1.47(0.76)	1.47(0.77)	0.0063
**LDL‐C (mmol/L)**	2.98(0.95)	3.08(0.95)	3.04(0.94)	2.92(0.94)	2.87(0.98)	<0.0001
**HDL‐C (mmol/L)**	1.39(0.42)	1.32(0.40)	1.37(0.41)	1.41(0.43)	1.44(0.44)	<0.0001
**ACM, *n* (%)**	1948(24.0)	242(11.9)	341(16.8)	540(26.6)	825(40.7)	<0.0001
**CVM, *n* (%)**	536(6.6)	67(3.3)	86(4.2)	136(6.7)	247(12.2)	<0.0001

*Notes*: Continuous variables were expressed as mean and standard deviation, while categorical variables were presented as the number of cases and the percentage of each category.

Abbreviations: ACM, all‐cause mortality; BMI, body mass index; CVM, cardiovascular mortality; DBP, diastolic blood pressure; FPG, fasting plasma glucose; HbA1c, hemoglobin type A1C; HDL‐C, high‐density lipoprotein cholesterol; LDL‐C, low‐density lipoprotein cholesterol; PP, pulse pressure; PPI, pulse pressure index; Q, quartile; SBP, systolic blood pressure; TC, total cholesterol; TG, total triglycerides;

### 3.2 Relationship Between PPI and Mortality in Patients With Hypertension

Over a median follow‐up of 98 months (interquartile range: 51–151 months), 1948 (24.0%) of the 8108 participants died, including 536 (6.6%) due to CVM and 1412 (17.4%) due to non‐CVM.

**FIGURE 2:**
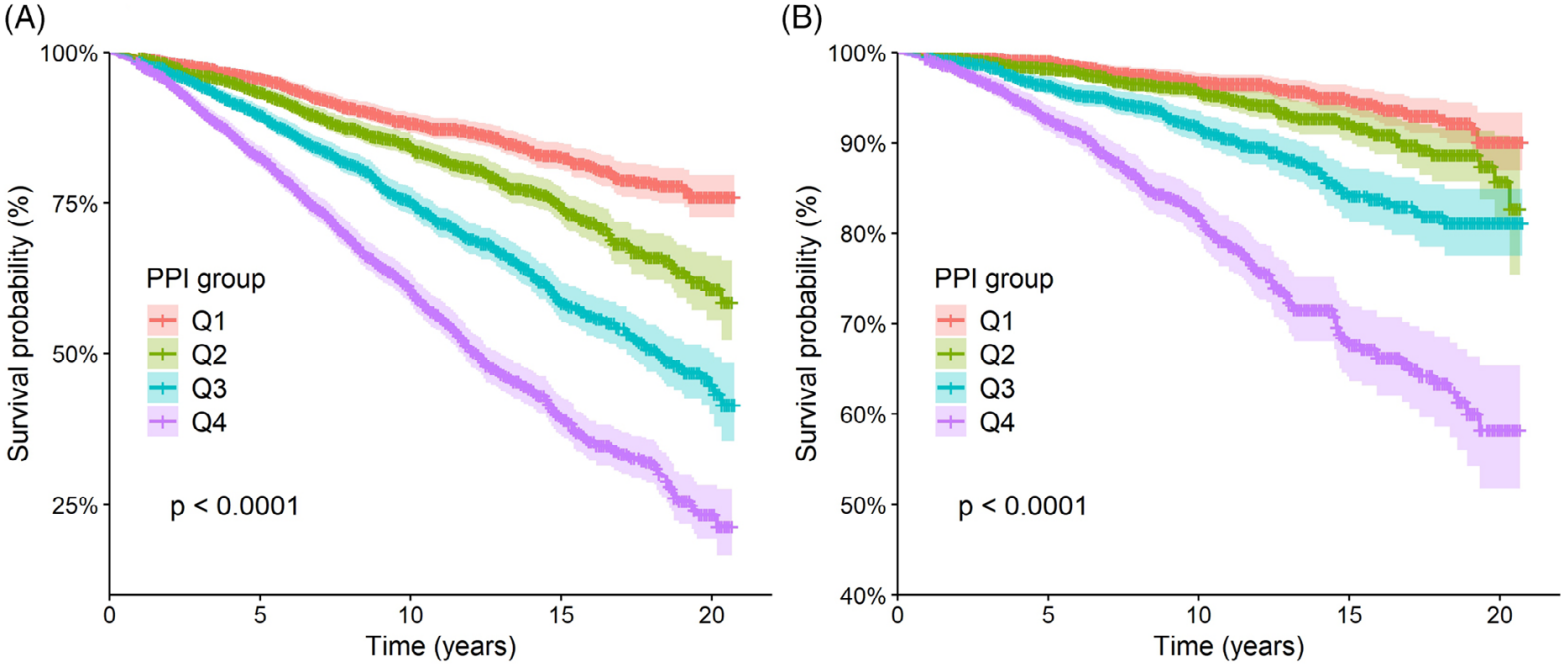
Kaplan–Meier curves of the survival rates for ACM and CVM in participants with hypertension according to the PPI quartiles. (A) All‐cause mortality; (B) Cardiovascular mortality.

Kaplan–Meier curves indicated differences in survival rates for ACM and CVM in patients with hypertension across PPI quartile groups (log‐rank *p* < 0.0001), with the fourth quartile group exhibiting the lowest survival rate (Figure 2A,B).

We developed three Cox proportional hazards regression models to explore the independent association of PPI with ACM and CVM in patients with hypertension. Across Models I to III, the trend test showed that PPI was associated with an increasing trend in ACM and CVM occurrence (all *p* for trend < 0.05) (Table 2). In Model III—after adjusting for age, sex, race, education level, BMI category, smoking history, drinking history, FPG, HbA1c, TC, TG, HDL‐C, and LDL‐C—the HR and 95% CI for ACM in the Q1, Q2, Q3, and Q4 groups were 1.000 (reference), 0.975 (0.783, 1.213), 1.070 (0.842, 1.359), and 1.336 (1.065, 1.675), respectively. Similarly, the HR and 95% CI for CVM in the Q1, Q2, Q3, and Q4 groups were 1.000 (reference), 0.930 (0.647, 1.337), 0.999 (0.724, 1.378), and 1.404 (0.985, 2.003), respectively (Table 2).

**TABLE 2:** Cox proportional hazards regression models on the relationships between PPI and both ACM and CVM in patients with hypertension.

Characteristic	Q1	Q2	Q3	Q4	*p* for trend
**ACM**					
Model I	Ref	1.509 (1.210,1.881)	2.817 (2.210, 3.591)	5.603 (4.533, 6.926)	<0.0001
Model II	Ref	0.981 (0.786,1.223)	1.097 (0.862,1.397)	1.409 (1.124,1.766)	0.0001
Model III	Ref	0.975 (0.783,1.213)	1.070 (0.842,1.359)	1.336 (1.065,1.675)	0.0011
**CVM**					
Model I	Ref	1.569 (1.100, 2.238)	3.089 (2.241,4.257)	7.972 (5.907, 10.758)	<0.0001
Model II	Ref	0.951 (0.662,1.367)	1.038 (0.756,1.424)	1.527 (1.080, 2.159)	0.0050
Model III	Ref	0.930 (0.647,1.337)	0.999(0.724,1.378)	1.404 (0.985,2.003)	0.0224

*Note*: Cox proportional hazards regression models were used to estimate the HR and 95% CI. Model I was unadjusted. Model II was adjusted for age, sex, race, education level, BMI category, smoking history, and drinking history. Model III was adjusted for age; sex; race; education level; BMI category; smoking history; drinking history; and FPG, HbA1c, TC, TG, HDL‐C, and LDL‐C levels.

Since the PPI value ranged from 0 to 1, we included PPI × 100 as a continuous variable in the Cox proportional hazards regression models. After adjusting for age, sex, race, education level, BMI category, smoking history, drinking history, FPG, HbA1c, TC, TG, HDL‐C, and LDL‐C, each 0.01 increase in PPI was associated with a 1.3% increase in the risk of ACM (HR = 1.013; 95% CI = 1.007–1.019; *p* = 0.001) and a 2.0% increase in the risk of CVM (HR = 1.020; 95% CI = 1.008–1.032; *p* = 0.001) (Table 3).

**TABLE 3:** Threshold effects analysis of the association of PPI with ACM and CVM in patients with hypertension.

	HR (95% CI)	*p*
**ACM**		
Fitting by the standard Cox proportional hazards regression model	1.013 (1.007–1.019)	0.001
Fitting by the two‐piecewise Cox proportional hazards regression model		
Inflection point	0.328	
PPI * 100 < 32.8	0.942 (0.904–0.982)	0.005
PPI * 100 ≥ 32.8	1.011 (1.005–1.016)	<0.001
*p* for Log‐likelihood ratio	0.003	
**CVM**		
Fitting by the standard Cox proportional hazards regression model	1.020 (1.008–1.03)	0.001
Fitting by the two‐piecewise Cox proportional hazards regression model		
Inflection point	0.363	
PPI * 100 < 36.3	0.933 (0.888–0.980)	0.006
PPI * 100 ≥ 36.3	1.019 (1.009–1.030)	<0.001
*p* for Log‐likelihood ratio	0.003	

*Note*: Cox proportional hazards regression models were used to estimate the HR and 95% CI. Adjusted for age, sex, race, education level, BMI category, smoking history, drinking history, and FPG, HbA1c, TC, TG, HDL‐C, and LDL‐C levels.

### 3.3 The Non‐Linear Relationships Between PPI and both ACM and CVM in Patients With Hypertension

**FIGURE 3:**
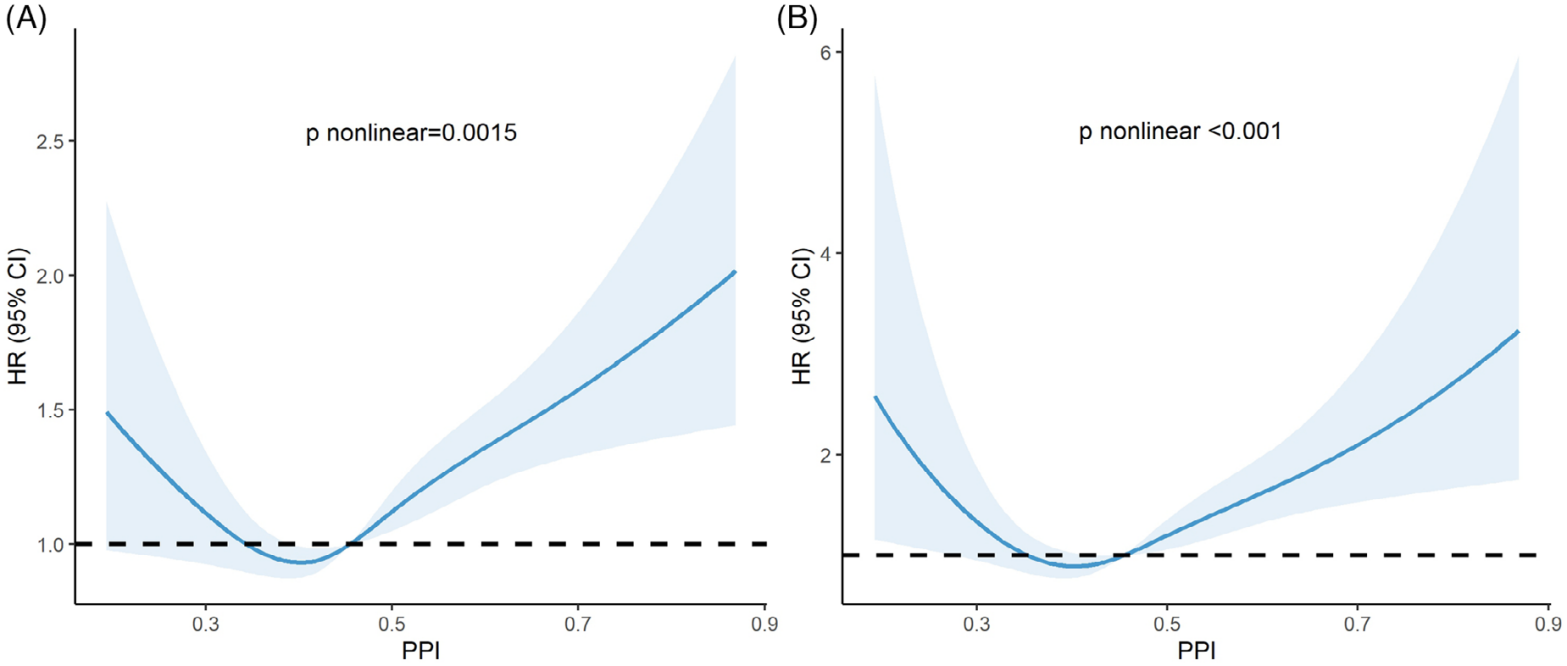
The U‐shaped curve relationships between PPI and mortality visualized by RCS. (A) All‐cause mortality; (B) Cardiovascular mortality. HR was adjusted for age, sex, race, education level, BMI category, smoking history, and drinking history, FPG, HbA1c, HDL, LDL, TG, and TC. Both *p* for non‐linear < 0.05.

RCS analysis was used to further explore the potential non‐linear associations of PPI with ACM and CVM. After adjusting for age, sex, race, education level, BMI category, smoking history, drinking history, FPG, HbA1c, HDL, LDL, TG, and TC, the results indicated that PPI exhibited a U‐shaped relationship with both ACM and CVM (all *p* for non‐linearity < 0.05) (Figure 3A,B).

Due to the non‐linear relationship between PPI and both ACM and CVM, as indicated by RCS, we conducted a threshold effects analysis and identified inflection points for ACM and CVM at PPI values of 0.327 and 0.363, respectively. Subsequently, we used PPI × 100 as a continuous variable and constructed two‐piecewise Cox proportional hazards regression models on either side of the new inflection points (32.8, 36.3) (both *p* values for log‐likelihood ratio < 0.05). After adjusting for age, sex, race, education level, BMI category, smoking history, drinking history, FPG, HbA1c, TC, TG, HDL‐C, and LDL‐C, we found that when PPI was lower than the inflection points, each 0.01 increase in PPI was associated with a 5.8% reduction in the risk of ACM (HR = 0.942; 95% CI = 0.904–0.982; *p* = 0.005) and a 6.7% reduction in the risk of CVM (HR = 0.933; 95% CI = 0.888–0.980; *p* = 0.006). Conversely, when PPI was higher than the inflection points, each 0.01 increase in PPI was associated with a 1.1% higher risk of ACM (HR = 1.011; 95% CI = 1.005–1.016; *p* < 0.001) and a 1.9% higher risk of CVM (HR = 1.019; 95% CI = 1.009–1.030; *p* = 0.001) (Table 3). Thus, PPI exhibited a U‐shaped association with ACM and CVM in patients with hypertension, with inflection points at 0.328 and 0.363, respectively.

### 3.4 Subgroup Analysis

We divided the participants into higher (ACM: PPI ≥ 0.328; CVM: PPI ≥ 0.363) and lower (ACM: PPI < 0.328; CVM: PPI < 0.363) PPI groups based on the PPI inflection points for ACM and CVM, respectively. Subgroup analysis was conducted to investigate the associations of PPI with ACM and CVM, according to age, sex, BMI category, smoking history, drinking history, and diabetes. Most subgroups did not exhibit significant interactions (*p* for interaction > 0.05), except for the age subgroup (ACM: *p* for interaction = 0.011; CVM: *p* for interaction = 0.008) and the diabetes subgroup (CVM: *p* for interaction = 0.009). Although age influenced the relationships between PPI and both ACM and CVM, these relationships were not statistically significant within subgroups (*p* > 0.05). In patients with diabetes, the risk of CVM was higher in the higher PPI group than that in the lower PPI group (HR = 1.78; 95% CI = 1.08–2.95; *p* = 0.025). However, this association was not observed in the nondiabetic participants (Table 4).

**TABLE 4:** Subgroup analysis of PPI groups and mortality in patients with hypertension.

	ACM				CVM			
		Higher PPI				Higher PPI		
Characteristics	Lower PPI	HR (95% CI)	*p*	*p* for interaction	Lower PPI	HR (95% CI)	*p*	*p* for interaction
Age				0.011				0.008
≤60	Ref	1.23 (0.92,1.62)	0.158		Ref	1.45 (0.92,2.29)	0.112	
>60	Ref	0.75 (0.51,1.11)	0.149		Ref	0.72 (0.43,1.20)	0.203	
Sex				0.411				0.373
Female	Ref	1.40 (0.95,2.04)	0.086		Ref	1.81 (1.05,3.14)	0.033	
Male	Ref	0.96 (0.73,1.25)	0.747		Ref	0.87 (0.59,1.29)	0.479	
BMI category				0.238				0.344
Normal weight	Ref	1.36 (0.85,2.18)	0.196		Ref	1.94 (0.96,3.96)	0.066	
Overweight	Ref	0.90 (0.58,1.38)	0.616		Ref	1.23 (0.67,2.26)	0.495	
Obesity	Ref	1.06 (0.78,1.45)	0.692		Ref	0.85 (0.55,1.33)	0.487	
Smoking history				0.429				0.322
NO	Ref	1.40 (0.93,2.12)	0.111		Ref	1.49 (0.8, 2.79)	0.213	
YES	Ref	1.03 (0.79,1.33)	0.842		Ref	1.00(0.69,1.45)	0.994	
Drinking history				0.287				0.245
NO	Ref	2.22 (1.09,4.52)	0.029		Ref	2.36 (1.04,5.37)	0.041	
YES	Ref	1.02 (0.81,1.29)	0.850		Ref	0.98 (0.69,1.38)	0.907	
Diabetes				0.060				0.009
NO	Ref	1.02 (0.78,1.33)	0.885		Ref	0.84 (0.56,1.27)	0.415	
YES	Ref	1.36 (0.92,2.02)	0.124		Ref	1.78 (1.08,2.95)	**0.025**	

*Note*: HR was adjusted for age, sex, race, education level, BMI category, smoking history, drinking history, FPG, HbA1c, TC, TG, and HDL‐C.

### 3.5 The Predictive Value of PPI and PP at Different Time Points for ACM and CVM in Patients With Hypertension

**FIGURE 4:**
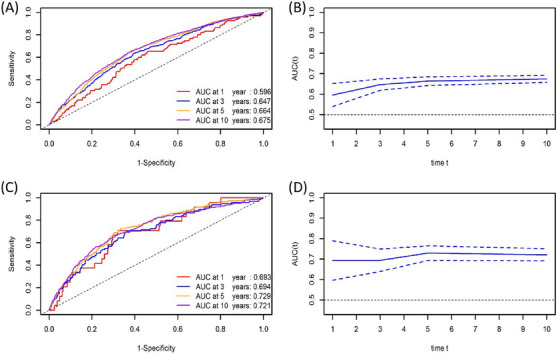
Time‐dependent ROC curves and AUC values of PPI for predicting ACM (A, B) and CVM (C, D).

Time‐dependent ROC curves were used to assess the predictive value of PPI for ACM and CVM in patients with hypertension at different time points. The results indicated that the AUCs of PPI for predicting ACM at 1, 3, 5, and 10 years were 0.596, 0.647, 0.664, and 0.675, respectively (Figure 4A,B). Similarly, the AUCs of PPI for predicting CVM at 1, 3, 5, and 10 years were 0.693, 0.694, 0.729, and 0.721, respectively (Figure 4C,D). These findings indicate that PPI exhibits good predictive value for both ACM and CVM, with this value gradually increasing within 5 years and then stabilizing afterward.

Furthermore, we compared the predictive value of PPI and PP for ACM and CVM at 1, 3, 5, and 10 years. The results indicated that the predictive value of PPI was higher than that of PP for ACM and CVM at 5 and 10 years (ACM: *p* = 0.036;, *p* < 0.001; CVM: *p* = 0.032, *p* < 0.001). However, there was no significant difference in the predictive value of PPI and PP at 1 and 3 years for ACM and CVM (all *p* > 0.05) (Table 5). Therefore, PPI exhibited a higher predictive value than PP at 5 and 10 years in patients with hypertension, whereas no significant difference was observed in the predictive value at 1 and 3 years.

**TABLE 5:** The predictive value of PPI and PP for ACM and CVM at different time points.

	PPI		PP		*p*
Characteristic	AUC	95% CI	AUC	95% CI	
ACM					
1 year	0.596	53.94–65.23	0.583	52.56–63.94	0.406
3 years	0.647	61.91–67.48	0.636	60.70–66.45	0.115
5 years	0.664	64.27–68.49	0.653	63.09–67.44	**0.036**
10 years	0.675	65.77–69.20	0.653	63.55–67.06	**0.000**
CVM					
1 year	0.693	59.69–78.97	0.642	53.62–74.70	0.099
3 years	0.694	64.01–74.89	0.674	61.52–73.21	0.107
5 years	0.729	69.36–76.50	0.710	67.10–74.81	**0.032**
10 years	0.721	69.14–75.03	0.700	67.01–73.05	**0.002**

*Note*: *p* represents the difference between PPI and PP of AUC at different time points.

## 4 Discussion

To the best of our knowledge, this is the first study to examine the association of PPI with ACM and CVM in patients with hypertension. The primary findings are as follows: First, PPI is an independent risk factor for ACM and CVM in patients with hypertension. PPI exhibits a U‐shaped curve with ACM and CVM occurrence, indicating that both significantly low and significantly high PPI levels may be associated with an increased risk of ACM and CVM, with the lowest risk observed at PPI values of 0.328 and 0.363. Second, PPI showed good predictive value for ACM and CVM occurrence in patients with hypertension at 1, 3, 5, and 10 years. Additionally, the predictive value of PPI for long‐term (5 and 10 years) ACM and CVM in patients with hypertension is higher than that of PP.

PP is an important indicator of arterial compliance [28]. As arterial stiffness increases with age, the amplitude of the forward pressure wave increases, and the premature arrival of wave reflections to the heart increases SBP and decreases DBP, resulting in a larger PP [29]. The pulsatile pressure from the heart is not properly buffered and is transmitted directly to target organs, leading to TOD [30]. Therefore, higher PP may be associated with poorer cardiovascular outcomes. Previous studies have shown that higher PP is associated with ACM (HR = 1.32; 95% CI = 1.28–1.36; *p* < 0.001), CVM (HR = 1.47; 95% CI = 1.40–1.55; *p* < 0.001), cardiovascular events (HR = 1.57; 95% CI = 1.55–1.59; *p* < 0.001), and other adverse outcomes, establishing it as an independent risk factor for ACM and CVM [31, 32]. Compared to SBP and DBP, PP has a higher predictive value for CAD development [33].

However, PP fluctuates with constant changes in blood pressure and is unrelated to the absolute value of blood pressure due to its “floating” characteristics. Consequently, the ability of the PP to reflect vascular compliance is somewhat limited [13]. PPI, which is the ratio of PP to SBP, overcomes the limitations associated with PP fluctuations; thus, it is a good indicator for assessing vascular compliance. The closer the PPI is to 1, the worse the vascular compliance.

PPI is closely associated with TOD in patients with hypertension. Left ventricular hypertrophy (LVH) is the most prevalent TOD in patients with hypertension, with 24 h PPI significantly higher in those with LVH than in those without LVH (*p* = 0.001) [34]. Additionally, PPI correlates with the estimated glomerular filtration rate, where patients with higher PPI exhibit poorer renal function and higher renal resistive index, thereby reflecting renal hemodynamics [35]. Previous studies have shown that PPI is significantly higher in rural populations who have experienced stroke than in those who have not, with stroke prevalence increasing with higher PPI [36]. Moreover, hypertension may induce vascular endothelial dysfunction through pathways such as oxidative stress and inflammation, promoting the development of atherosclerosis [37]. Assessment of coronary artery stenosis severity in patients with CAD using the coronary artery disease‐reporting and data system (CAD‐RADS) score revealed that the PPI in the subgroup with CAD‐RADS scores of 3–5 was significantly higher than that in the subgroup with CAD‐RADS scores of 0–2 (0.419 ± 0.078 vs. 0.388 ± 0.072, *p *< 0.001) [38]. Measurement of carotid intima‐media thickness (CIMT) in patients with hypertension showed that PPI was significantly higher in the group with elevated CIMT than in the control group. After adjusting for traditional risk factors, only PPI emerged as an independent risk factor for elevated CIMT (OR = 1.644, 95% CI = 1.280–2.112, *p* < 0.001). Additionally, the predictive value of PPI for increased CIMT was stronger than that of PP (AUC = 0.664 vs. 0.591, *p *= 0.006) [39]. Therefore, higher PPI levels in patients with hypertension may reflect more severe TOD, such as the heart, brain, kidneys, and blood vessels. Consequently, an elevation in PPI indicates an increased risk of cardiovascular events, subsequently leading to an increased risk of CVM and ACM.

Our study revealed a novel finding: the relationship between PPI and mortality in patients with hypertension follows a U‐shaped curve. Lower PPI levels are also associated with increased mortality; however, the mechanisms underlying poor prognoses differ between low and high PPI patients. The baseline characteristics suggest that patients with high PPI are predominantly elderly, exhibiting elevated SBP and PP, with PP showing a more significant increase and leading to a high PPI. In contrast, patients with low PPI are primarily middle‐aged, characterized by a significant increase in DBP, no significant increase in SBP, low PP, and consequently low PPI. The difference in age and blood pressure characteristics in patients with varying values of PPI may be due to the distinct changing trends of the three components of blood pressure (SBP, DBP, and PP) with an increase in age. SBP shows a continuous upward trend from 30 to 84 years of age, while DBP remains unchanged or declines after 50–60 years of age. Therefore, with an increase in age, the rise in SBP is larger than that of DBP, leading to a gradual increase in PP with an increase in age [40]. Therefore, we believe that the increased mortality risk in patients with lower PPI is primarily due to elevated DBP. The underlying hemodynamic mechanism driving DBP elevation is an increase in systemic vascular resistance. Higher DBP forces the heart to overcome greater resistance during ejection, increasing the burden on the heart and leading to ventricular hypertrophy and impaired heart function [41, 42]. This may be an important mechanism for DBP elevation, increasing cardiovascular death and the risk of all‐cause mortality.

Although both PPI and PP are reliable indicators of vascular compliance, PPI not only reflects the dynamic compliance of blood vessels, but also reflects the inherent compliance of blood vessels. Therefore, PPI may be a better indicator of vascular compliance, as PPI is better than PP in assessing cardiovascular outcomes [13]. As Karadavut et al. [43] reported, PPI showed a greater predictive value of CAD progression than PP (AUC = 0.649 vs. 0.574, *p *= 0.03). However, no previous studies have compared the effects of PPI and PP on mortality in hypertensive patients at varying time points. This study addressed this gap by using time‐dependent ROC curves to further compare the predictive value of PPI and PP on ACM and CVM at varying time points (1, 3, 5, and 10 years). The results showed that PPI outperformed PP in predicting ACM and CVM at 5 and 10 years, suggesting that PPI may have an advantage in predicting long‐term mortality (5 and 10 years) in hypertensive patients. A possible explanation for this difference is that the predictive value of PPI and PP for the mortality of hypertensive patients changes over time. The AUC values for PPI and PP in predicting ACM and CVM gradually increased over the first 5 years and stabilized after 5 years. This suggests the superiority of PPI over PP as a predictor of long‐term (5‐ and 10‐year) mortality in hypertensive patients.

The main strength of this study is that the data came from the NHANES, which provides an adequate sample size and a long follow‐up period, thus supporting reliable conclusions. However, this study has some limitations. First, most participants were taking antihypertensive medications, but we could not determine whether these medications influenced PPI. Second, this study focused on patients with hypertension in the USA, which may limit the generalizability of the findings to other populations. Third, despite adjusting for many covariates, there may have been other unknown factors influencing the results. Fourth, PPI does not adequately describe the oscillation of PP over the mean value in subjects with different mean blood pressure; defining PPI as PP/MAP may better describe this. We will closely monitor the correlation between the new PP index (PP/MAP) and mortality in patients with hypertension in subsequent studies. Finally, due to the cross‐sectional nature of the NHANES, we could not establish whether controlling for PPI reduces mortality in patients with hypertension.

## 5 Conclusion

Our findings indicate that PPI is an independent risk factor for the development of ACM and CVM in patients with hypertension and shows a U‐shaped association with these outcomes. Additionally, PPI has a higher predictive value than PP for the development of ACM and CVM at 5 and 10 years. Therefore, PPI, as a noninvasive indicator obtainable through routine blood pressure measurements, can be used to identify patients with hypertension at higher risk of mortality and guide more aggressive antihypertensive treatment strategies. However, clinical guidelines have not yet recognized PPI as a target for intervention in patients with hypertension. Further clinical studies are necessary to explore the potential mechanisms through which PPI influences mortality in this population.

## Author Contributions

Conception and design: Hongjin Jin, Shuo An; Methodology: Hongjin Jin, Shusheng Fang; Writing–Original Draft Preparation: Hongjin Jin; Writing–Review & Editing, Hongjin Jin, Yanchun Ding; All authors read and approved the final manuscript.

## Ethics Statement

The NHANES was approved by the National Center for Health Statistics Research Ethics Review Board, and all participants have provided written informed consent.

## Conflicts of Interest

The authors declare no conflicts of interest.

## Data Availability

The datasets supporting the conclusions of this article are available in the NHANES repository, https://wwwn.cdc.gov/nchs/nhanes/Default.aspx.
